# Radiotriquetral Ligament in Madelung’s Deformity Associated with Leri-Weill’s Dyschondrosteosis

**DOI:** 10.7759/cureus.7100

**Published:** 2020-02-25

**Authors:** Alessandro De Leucio, Sybille Castelein, Michel Bellemans, Paolo Simoni

**Affiliations:** 1 Radiology and Medical Imaging, Queen Fabiola Children's University Hospital, Brussels, BEL; 2 Orthopedics and Traumatology, Queen Fabiola Children's University Hospital, Brussels, BEL

**Keywords:** madelung’s deformity, leri-weill's dyschondrosteosis, vickers ligament, radiotriquetral ligament, mri, wrist, radiography

## Abstract

Madelung's deformity (MD) is frequently associated with Leri-Weill's dyschondrosteosis (LWD) even if the primary isolated form (PI-MD) is much more common.

Recent studies pointed out how two abnormal ligaments, the Vickers ligament (VL) and the radiotriquetral ligament (RTL), are defining traits of MD. To date, in PI-MD, both VL and RTL have been reported. In MD associated with LWD (LWD-MD), the VL is also present, but the RTL has never been reported.

We herein report the first case of MD associated with a genetically confirmed LDW with an RTL, detected on MRI.

This report describes the MRI imaging features of MD-LWD, which have not been adequately characterized in previous literature.

## Introduction

Madelung's deformity (MD) is a generic term encompassing different pathological conditions, including primary isolated MD (PI-MD), MD associated to Leri-Weill's dyschondrosteosis (LWD-MD), and several mimicking conditions defined "pseudo-MD" including post-traumatic and post-infective forms, forms associated to Turner's syndrome, multiple hereditary exostoses, and Ollier disease [1].

A supernumerary volar extrinsic ligament of the wrist, the so-called "Vickers ligament" (VL), allows distinguishing MD from "pseudo-MD" in which the VL is not visualized [1-3]. In LWD, the carpal abnormities of MD coexist with mesomelia and short stature, defining a rare autosomal dominant inherited condition with unknown prevalence [4-5].

Vickers ligament is considered an abnormally short and thickened radiolunate ligament. Unlike the normal radiolunate ligament originates from the epiphysis of the distal radius, the VL rises more proximally from the radial metaphysis, creating a tether across the volar-ulnar physis that restricts growth across this segment, resulting in a "V" shaped deformity of the whole distal radio-ulnar joint surface [2, 3, 6]. More recently, another anomalous volar ligament just lateral to the VL, defined "radiotriquetral ligament" (RTL), was described in MD. Cook first mentioned RTL in 1996 [7], but its appearance was comprehensively assessed on MRI by Stehling using a 3-Tesla unit with isotropic images [8].

Recent studies have suggested that RTL in the same way as VL is a defining element of MD, not present in pseudo-MD [9].

The role of RTL in the pathogenesis of MD is still unclear [8].

## Case presentation

An eight-year-old girl was referred to the pediatric orthopedic surgeon for mild pain at her right wrist experienced while writing.

Clinical examination revealed a bilateral and symmetrical dorsal protrusion of ulna ("bayonet sign"), and radial bowing. A mild bilateral radio-ulnar instability with normal wrist prono-supination and flexo-extension were also noted in the clinical records. Both the weight and height of the patient were below the 10th percentile.

**Figure 1:**
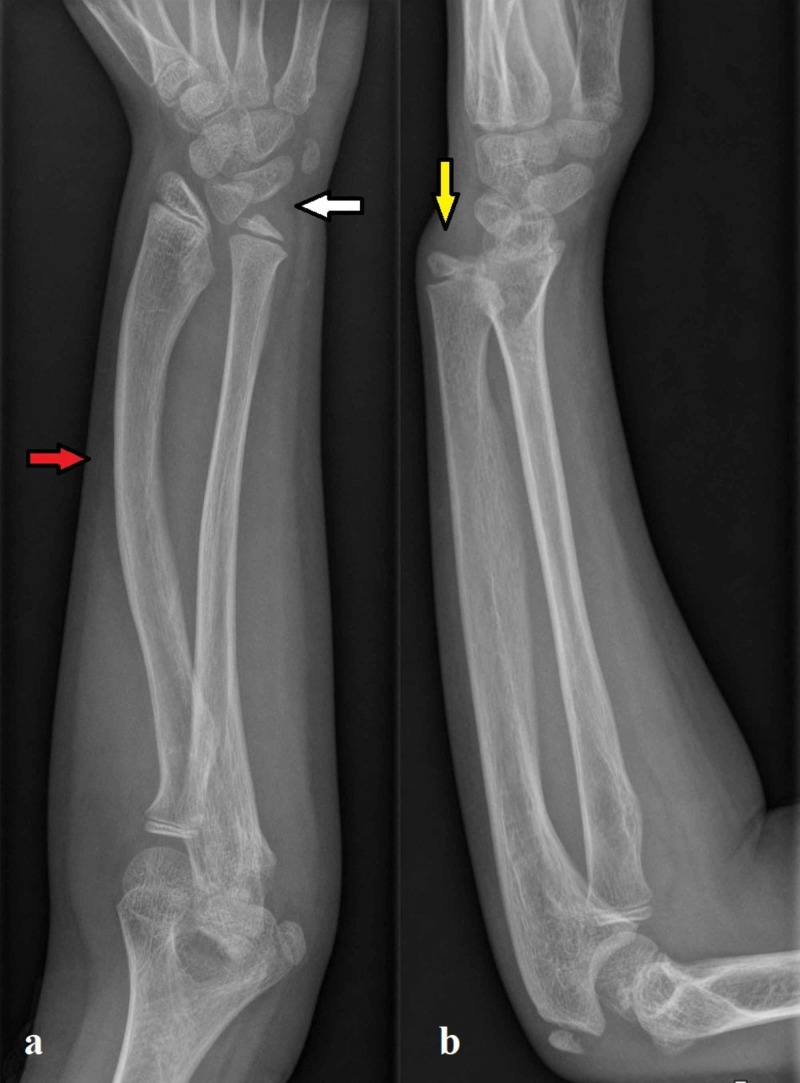
Radiography. The anteroposterior radiograph (a) of the left wrist shows an increased radial inclination (~ 32°) with the deformation of the carpus that acquired a triangular appearance (white arrow). Shortening and bowing of the radial diaphysis (red arrow) are also evident. The lateral radiograph (b) shows the volar tilt of the distal radius with an angular measure at the upper limit of normality (~ 21°). Dorsal subluxation of the ulnar head (yellow arrow) is also present.

**Figure 2:**
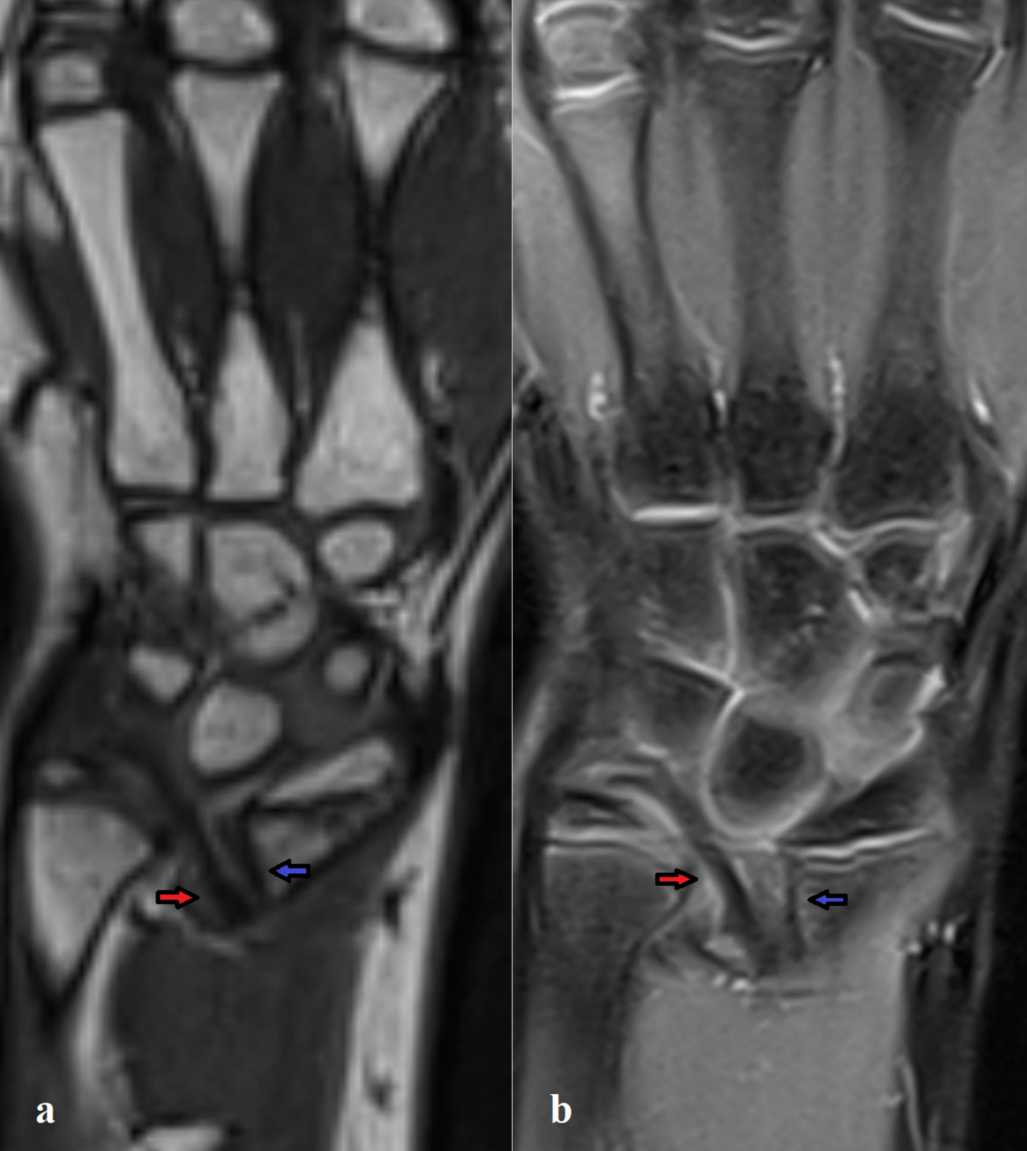
MRI aspect of the VL and RTL. Visualization of VL (blue arrow) and RTL (red arrow) on T1-space isotropic coronal MRI (a) and PD-tse fat suppressed coronal MRI (b). VL appears as an abnormal volar radiolunate ligament short and thickened. RTL is another abnormal volar ligament long and thickened just lateral to the VL. VL, Vickers ligament; RTL, radiotriquetral ligament; PD-tse, proton density-turbo spin echo

**Figure 3:**
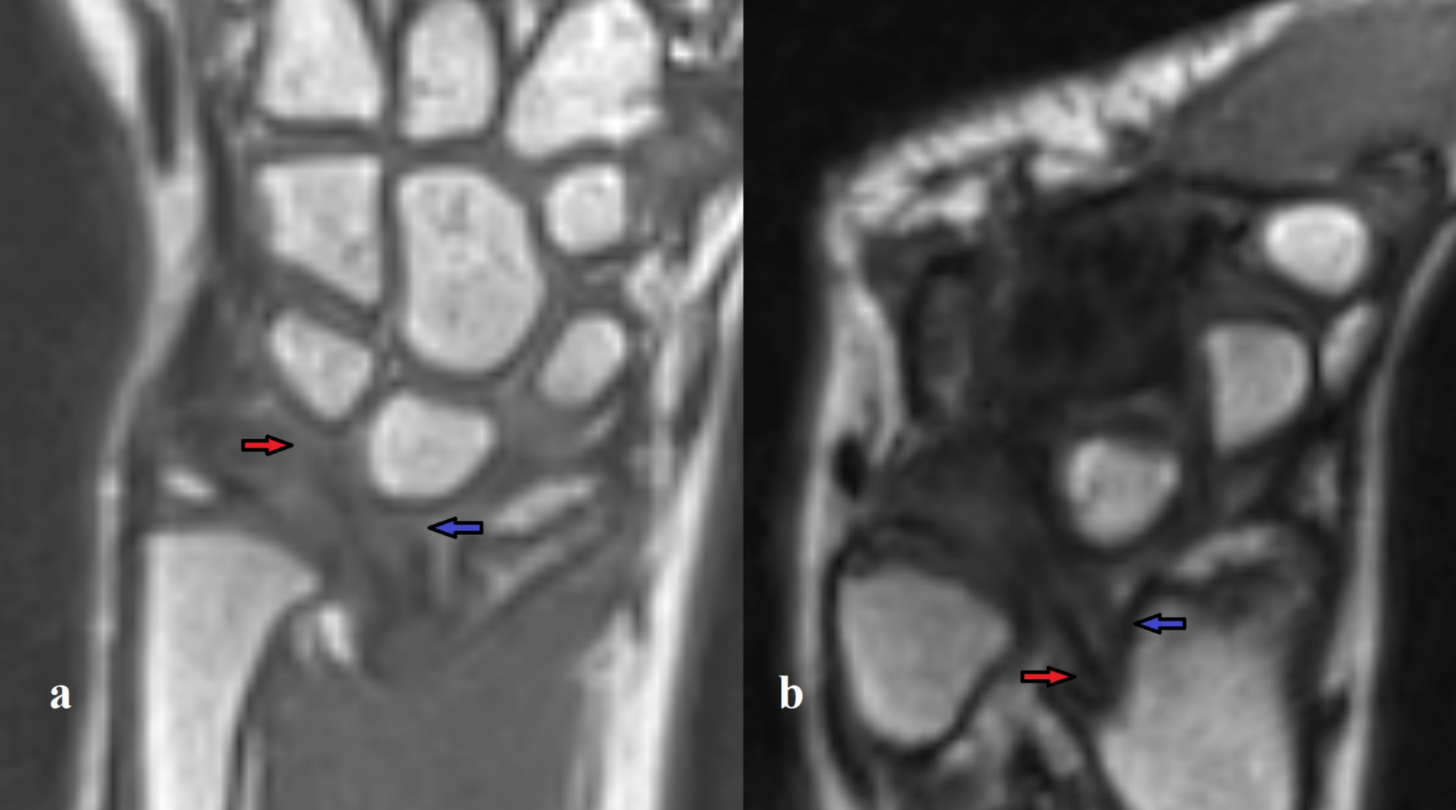
Insertions of the VL and the RTL. (a) T1-space isotropic coronal MRIs of RTL trichetral insertion (red arrow) and VL lunar insertion (blue arrow); (b) T1-space isotropic coronal MRIs of RTL (red arrow) and VL (blue arrow) radial origins. VL, Vickers ligament; RTL, radiotriquetral ligament.

Radiographs of her right wrist showed an increased angle of radial inclination (~ 32°) on the anteroposterior radiograph of the distal radius and volar tilt at the upper limit of normality (~ 21°) on the lateral radiograph. Radiographs of the corresponding right forearm showed a shortening and bowing of the radial diaphysis, a dorsal subluxation of the ulnar head along with a triangular appearance of the carpus (Figure 1). The radiographic examination of the controlateral wrist and forearm confirmed the presence of the same appearance. An MRI of her right wrist allowed to confirm the diagnosis by the presence of VL and RTL (Figures 2-3).

Genetic analysis revealed a deletion of the X chromosome (Xp22.33) located between the PLCXD1 and the SHOX (SHort stature HOmeoboX gene), confirming the diagnosis of LDW.

At the follow-up, the pain vanished a few days after the administration of nonsteroidal anti-inflammatory drug (NSAID) and the patient was treated conservatively. Growth hormone therapy was started because of the growth retardation.

## Discussion

The presence of the VL and RTL in PI-MD and its absence in pseudo-MD was solidly proven [1, 8-10].

Our case confirmed with MRI evidence the presence of these abnormal ligaments in a patient with a genetically confirmed LDW.

In 2018, Hanson published the most extensive series of classic MD studied at MRI. In his series of eight cases of MD, the RTL is a defining trait of PI-MD, while it is never present in pseudo-MD. However, there was no LWD-MD reported in this series [9].

None of the other smaller studies focused on RTL demonstrated the presence of this ligament in patients with LWD [11-12].

Cook [7] and Stehling [8] reported the presence of the RTL in bilateral MD probably associated with LWD. Nevertheless, their reports lacked confirmation by genetic analysis.

In contrast, Van Zwieten postulated that the RTL is present only in PI-MD [10]. Our case is not in keeping with this evidence suggesting that both VL and RTL can be found in PI-MD and in LWD-MD.

The visualization of VL and RTL is essential in the differential diagnosis because they are not present on conditions mimicking MD, the so-called pseudo-MD [1, 8-10]. Hence, the visualization of these ligaments can narrow the differential diagnosis when coping with the abnormal appearance of the radio-carpal joint.

## Conclusions

We herein report the first case in the medical literature of RTL in a patient with MD associated with an LWD confirmed by genetic analysis.

Our case confirms that high-resolution MRI imaging is mandatory to visualize this abnormal ligament thanks to the multiplanar reconstruction to depict the origin, insertion, and appearance of these abnormal structures (Figures 2-3).

More extensive series, ideally by a multicentric study due to the relative rarity of MD could shed light on the relationship between imaging findings, genetic features, and associated pathology in MD.
